# Effect of Exogenous Auxin Treatment on Cell Wall Polymers of Strawberry Fruit

**DOI:** 10.3390/ijms22126294

**Published:** 2021-06-11

**Authors:** Ricardo I. Castro, Ana González-Feliu, Marcelo Muñoz-Vera, Felipe Valenzuela-Riffo, Carolina Parra-Palma, Luis Morales-Quintana

**Affiliations:** 1 Multidisciplinary Agroindustry Research Laboratory, Facultad de Ingeniería, Instituto de Ciencias Químicas Aplicadas, Universidad Autónoma de Chile, Talca 3467987, Chile; ricardo.castro@uautonoma.cl; 2 Carrera de Ingeniería en Biotecnología, Universidad Católica del Maule, Talca 3460000, Chile; anafeliu11@gmail.com; 3 Multidisciplinary Agroindustry Research Laboratory, Talca 3467987, Chile; marcelo.uatalca@hotmail.com; 4 Programa de Doctorado en Ciencias Mención Ingeniería Genética Vegetal, Instituto de Ciencias Biológicas, Universidad de Talca, Talca 3465548, Chile; felipe.v.r.89@gmail.com (F.V.-R.); cparra@utalca.cl (C.P.-P.); 5 Multidisciplinary Agroindustry Research Laboratory, Facultad de Ciencias de la Salud, Instituto de Ciencias Biomédicas, Universidad Autónoma de Chile, Talca 3467987, Chile

**Keywords:** auxin inhibitor, auxin treatment, cell wall polymer, strawberry fruit, thermogravimetry analyses

## Abstract

The role of auxin in the fruit-ripening process during the early developmental stages of commercial strawberry fruits (*Fragaria x ananassa*) has been previously described, with auxin production occurring in achenes and moving to the receptacle. Additionally, fruit softening is a consequence of the depolymerization and solubilization of cell wall components produced by the action of a group of proteins and enzymes. The aim of this study was to compare the effect of exogenous auxin treatment on the physiological properties of the cell wall-associated polysaccharide contents of strawberry fruits. We combined thermogravimetric (TG) analysis with analyses of the mRNA abundance, enzymatic activity, and physiological characteristics related to the cell wall. The samples did not show a change in fruit firmness at 48 h post-treatment; by contrast, we showed changes in the cell wall stability based on TG and differential thermogravimetric (DTG) analysis curves. Less degradation of the cell wall polymers was observed after auxin treatment at 48 h post-treatment. The results of our study indicate that auxin treatment delays the cell wall disassembly process in strawberries.

## 1. Introduction

Auxin has been described as important for fruit growth, playing notable roles from flower formation to fruit ripening [1,2]. At the early stage of fruit development, auxin is believed to participate in the cell expansion associated with fruit growth [3,4]. During the last ripening stages, a role has also been proposed [2,3,4], and different studies have tried to clarify this role, although the cellular and molecular processes involved are not yet clear. For example, the role of auxin in the ripening of strawberry (*Fragaria x ananassa*) fruits has been limited to the early stages of development, in which the delivery of auxin from achenes is responsible for receptacle growth [5]. At later stages (75% or 100% ripe), during receptacle growth, other phytohormones have been shown to participate to different degrees, from the general involvement of gibberellins [6] to abscisic acid [6,7,8,9,10,11,12,13,14,15,16].

*Fragaria x ananassa* (commercial strawberry) is an economically important fruit species that is consumed around the world for its pleasant aroma, flavor, and nutritional value [17,18]. Strawberries make up 241,000 ha of crop area with a fruit production of 4.5 million tons in 2012, and with a constant increase showing 8.1 million tons in 2014, while in 2019, global strawberry production reached 8.9 million tons in 396,401 ha estimated in the world (http://www.fao.org/home/en/, accessed on 4 June 2021). However, the primary limitations of this fruit are its very short postharvest life with elevated economic losses, which limits the postharvest life of the fruit [19,20]. In the strawberry fruit, texture is an important attribute for consumer acceptability, and it is related with fruit softening during ripening and postharvest [21]. In this form, strawberry fruit exhibits a rapid softening during ripening [18,19]. Thus, fruit softening is partly explained by cell wall breakdown [21].

Plant cell walls are the most abundant source of organic carbon on the planet and have a fundamental role in the structural stability of the plant cell [22]. At the structural level, the plant cell walls are a dynamic and complex supra-molecular structure formed by crystalline cellulose microfibrils surrounded by an amorphous matrix of polysaccharides such as pectins and hemicellulose, proteins, and inorganic molecules [23,24,25]. The cell wall provides mechanical support and cellular shape, regulates the development and growth of the cells of fruits and vegetative tissues, and is the first and most important barrier against different abiotic and biotic stresses [21,26,27]. The cell wall structure is modified by the coordinated action of several enzymes that can lead to the dissolution of the middle lamella, decrease cell wall strength and cell-to-cell adhesion, and change in polysaccharide solubilization and depolymerization in fruits, resulting in an increase in softening during ripening [16,27,28,29,30,31].

The cell wall disassembly process occurs principally during the final step of fruit ripening and has been researched using different techniques in most fleshy fruits, including strawberries, and auxin apparently plays an important role in the early ripening stages. Although it has been well studied at the molecular level using biochemical and molecular biology techniques, there is still a lack of understanding of the physicochemical basis through which auxin is involved in cell wall changes in strawberry fruits. For this reason, the objective was to evaluate the thermal properties of the cell wall disassembly process after treatment with auxin or 2,3,5-triiodobenzoic acid (TIBA), which is an auxin transport inhibitor. In this paper, we used thermogravimetric analysis (TGA) combined with a DTG curve, biochemical analysis (evaluating total enzymatic activity), molecular biology analysis (evaluating the mRNA abundance of principal cell wall-related genes), and quality fruit (parameter such as firmness, weight, and soluble solid concentration) analyses related to cell wall remodeling.

## 2. Results and Discussion

### 2.1. Fruit Quality Determinations

Fruit development is an irreversible process that ends with the ripe stage of the fruit; this process alters the cell wall architecture when it is possible to divide it into two phases (cell division phase and cell expansion phase) in most fruits [16]. By contrast, in strawberries, the two phases make it difficult to establish the functional separation of these phases [32], because in the strawberry, the development and ripening process are two events that occur in parallel [33]. Additionally, different plant hormones control the development or cell division phase (I phase), and other hormones can control the phase II or expansion phase during fruit ripening [6]. Relatedly, the role of the hormone auxin in the development or ripening fruit processes in strawberry fruits (*F. x ananassa*) corresponds to the early development stages when auxin production occurs in the achenes and moves to the receptacle [6,34].

Auxin is involved in a number of growth and developmental processes and has a prominent role in the acid growth during cell expansion [35,36,37]. Recently, IPA-N3 (an active auxin analog) was used as an auxin tracer and provided in vitro evidence of the presence of auxin binding sites in the cell walls of elongating cells in Arabidopsis [38]; however, no evidence has been described in strawberries.

To determine the effect of auxin on the ripening and quality of strawberry fruits, we applied exogenous auxin (Aux) and evaluated different physiological parameters related to texture and fruit softening changes, which are directly related to cell wall remodeling [39,40,41,42,43,44,45,46].

Symons et al. (2012) [6] classified the development and ripening of strawberries into six different stages after the flower stage, and they observed that auxin was found in the small green (SG; 11–12 post-anthesis days) stage and with a maximum peak concentration at the large green (LG; 16–17 post-anthesis days) stage, while during the small white (SW; 18–19 post-anthesis days) stage, the concentration decreased to basal or zero auxin levels [6]. Coincidentally, firmness is reduced primarily after these stages, between the SW and the large white stage (LW; 20–23 post-anthesis days) [15]. These changes in firmness rate have been found to differ with different levels of softening, and the transition between the white and 25% ripe or 50% ripe stages occurs when the greatest loss of firmness occurs [18,32,34,35,36,37,38,39,40,41,42,43,44,45,47], although the process begins in the SW to LW. From these studies, we decided to use the SW stage, since it is the last stage prior to the greatest firmness reduction of the fruit [15,44]; this choice is concordant with previous studies that determined the white (W) stage as optimal for studies on the hormonal effects in strawberries [15,16,48,49,50,51].

**Figure 1:**
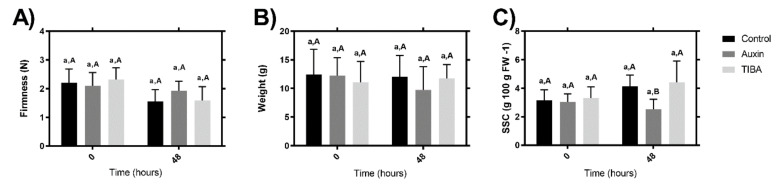
Changes in fruit the quality parameters related with cell wall modifications. (**A**) Fruit firmness, (**B**) weight, and (**C**) SSC. Different lowercase letters indicate significant differences in each treatment (control, auxin, or TIBA) over two evaluated times (0 and 48 h). Different capital letters indicate significant differences between the three treatments at two times. Differences between means ± standard errors (SE) (*n* = 15) were determined by ANOVA and the LSD test (*p <* 0.05).

Fruit firmness of the auxin-, TIBA-, or control-treated fruit was determined. The results showed that there were no significant changes between the different samples analyzed, it was comparing the result of the samples 48 h with respect to 0 h post-treatment (Figure 1A); similarly, there were no significant differences at each time point, particularly between the two treatments with respect to the control samples (Figure 1A). Previously, Chen et al. (2016) [32] described a similar result after two days of auxin treatment of ‘Akihime’ strawberries; specifically, the authors showed that exogenous auxin treatment of strawberries had a slight effect on the fruit color but not on fitments [32]. With respect to the fruit weight, Figure 1B shows no significant differences between the auxin, TIBA, or control treatments, and independent of the time and sample, the values were all between 8 and 16 g (Figure 1B). Aux treatment produced a decrease in the SSC values compared to the control or TIBA treatment at 48 h post-treatment; however, no significant differences were observed between the Aux treatments at 0 h with respect to 48 h (Figure 1C).

### 2.2. Thermal Stability Analysis of the Treated Fruits

**Figure 2:**
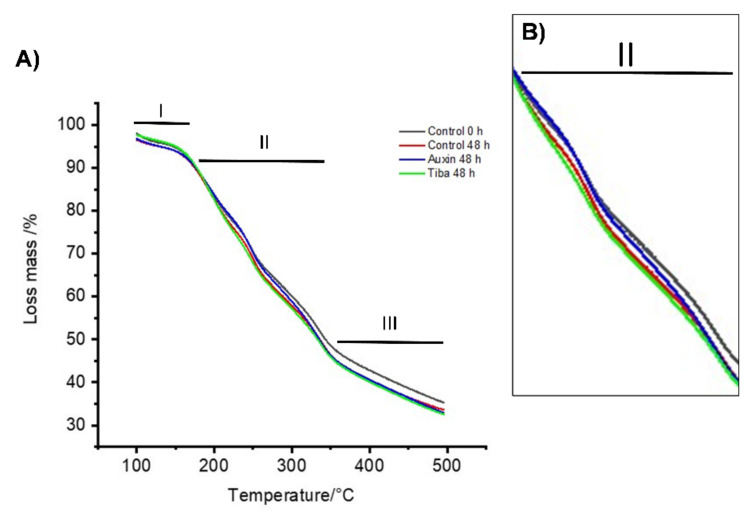
Thermogravimetric analysis (TGA). (**A**) Thermograms derived from each treatment at two different times with temperatures between 100 and 500 °C. (**B**) Zoom in on region II of the thermogram where the main differences between treatments are observed.

Fruit firmness is evaluated using a macroscopic method, and perhaps for this reason, it is not possible to observe differences after the treatment (Figure 1A), even when other authors have indicated that auxin has an effect on gene expression and cell wall-related enzyme activity [34,48,52,53]. For this reason, it was necessary to use an alternative methodology to determine the effect of auxin on the cell wall component after exogenous treatment. Recently, we performed a study on the changes in the physiological properties of strawberry fruit and showed lower thermal stability at the ripe stage than at the green stage [33], and the same methodology was used to evaluate the changes in the cell wall thermal stability produced by ABA treatment [16]. Thus, the integrity and stability of the polymeric components that form the cell walls in strawberry fruits were evaluated through thermal stability assays (Figure 2). Dry samples derived from three experimental groups at two time points were used for thermogravimetric analysis. Figure 2 shows the graphic with TGA curves. The thermograms were divided into three phases or regions according to Castro and Morales-Quintana (2019) [18]. In the graphic, the region of temperatures between 100 and 180 °C is named as region I or the initial phase. In this region, we observe no significant differences in the TGA curves between the control 0 h, control at 48 h, and the TIBA at 48 h (Figure 2A). However, a slight difference between 48 h after auxin treatment with respect to the other three samples was observed (Figure 2A). Additionally, the initial phase described that all samples had greater thermal stability (Figure 2A), which is similar to what has been described previously for different strawberry cultivars such as ‘Camarosa’ [18], ‘Cristal’, or ‘Portola’ [33], or Chilean strawberry [54]. The second region in the graphic is named the intermediate phase or region II and comprised temperatures between 200 and 350 °C. The result showed that the auxin 48 h and control 0 h treatments did not exhibit differences between them (Figure 2B), and more importantly, it resulted in greater thermal stability values than the control 48 h and TIBA 48 h treatments (Figure 2B). The third region is named as the final phase or region III, and corresponding to temperatures between 350 and 500 °C, the analysis showed that the control 0 h treatments showed greater thermal stability values than the control 48 h, auxin 48 h, and TIBA 48 h treatments (Figure 2A).

**Figure 3:**
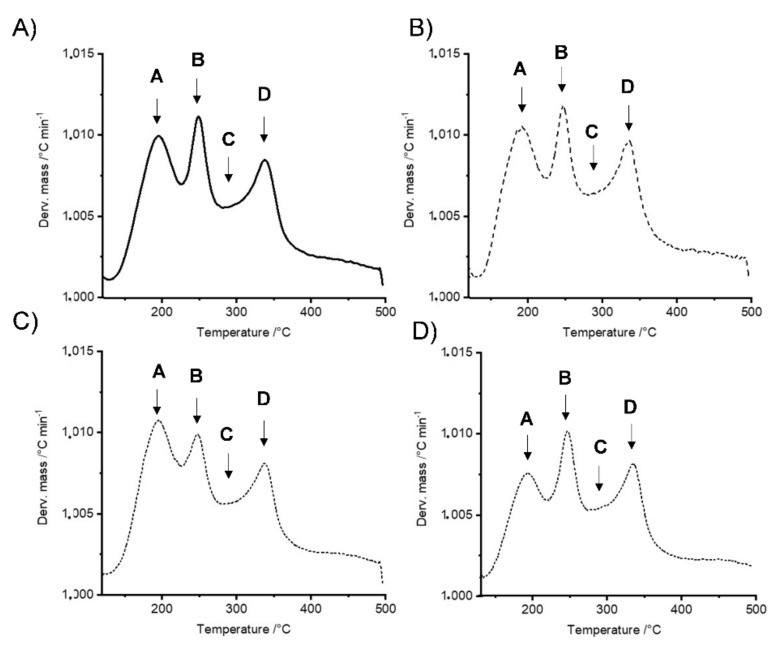
TG/DTG thermogram. (**A**) Control treatment at 0 h; (**B**) control treatment at 48 h; (**C**) auxin treatment at 48 h; (**D**) TIBA treatment at 48 h.

Recently, we showed that differential thermogravimetric analysis (DTG), which is obtained from the first derivative of the TGA values, can be divided into four maximum degradation peaks [16,33,53,54]. Figure 3 shows the four possible regions for these peaks. First, peak A did not show any difference between the control 0 h sample (Figure 3A) and the control 48 h sample (Figure 3B), while the auxin-treated 48 h sample (Figure 3C) showed a slightly higher value than the 1.010 value for the control at 0 h. In turn, the sample treated with TIBA for 48 h (Figure 3D) showed a value slightly lower than that value. This is an interesting result because in the A region, it is possible to observe the decomposition of compounds (principally carbohydrates such as xylose and glucose) related to the three principal polymer compounds that form the cell wall (cellulose, hemicelluloses, and pectin) [33,53,54,55]. The B region principally showed a decomposition of hemicellulose fractions between temperatures of 200 and 300 °C [56] and shorter chain pectin fractions at approximately 250 °C [56] (Figure 3). Figure 3 shows that only auxin 48 h post-treatment had values lower than 1.010, while the other three treatments did not display any differences (Figure 3). Finally, region C primarily shows the depolymerization of hemicelluloses (region C) [33,53,57,58], and region D indicates temperatures between 360 and 400 °C that were associated with the decomposition of lignin and cellulose [33,53,57,58,59]. These two regions showed similar tendencies in the sample profiles, and no differences were observed (Figure 3). This result was similar to that described previously for strawberries treated with ABA or fluoridone [16].

**Figure 4:**
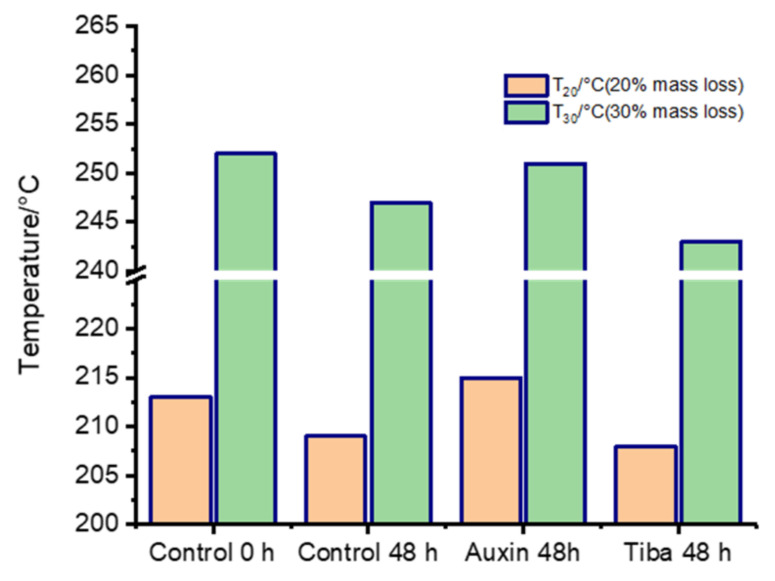
Temperature degradation of different samples 48 h post-treatment compared to control 0 h post-treatment. The graph shows the temperatures corresponding to sample loss of 20% of 30% mass.

Additionally, Figure 4 shows the temperatures required to obtain 20% and 30% mass loss in the three samples at 48 h post-treatment with respect to the control sample at 0 h post-treatment. Accordingly, the control-treated samples at 48 h post-treatment showed a similar value to that of the TIBA samples 48 h post-treatment in the temperature required to achieve 20% mass loss (209 and 208 °C, respectively), while the control 0 h and auxin 48 h post-treatment samples showed temperatures of 213 and 215 °C, respectively, showing that auxin needs a greater temperature to obtain a similar degradation degree, indicating more thermal stability (Figure 4). With respect to the temperature needed to lose 30% fruit mass, the control sample at 0 h showed a temperature of 252 °C, while for the auxin-treated samples at 48 h post-treatment, a temperature of 251 °C was necessary (Figure 4). Thus, the auxin-treated sample was similar to the control samples at 0 h, showing the highest temperature compared to the control- or TIBA-treated samples at 48 h post-treatment, which showed temperatures of 246 and 242 °C, respectively, indicating that the polymer fragments of the samples subjected to TIBA treatment at 48 h post-treatment were more depolymerized or more fragmented than those of the other three samples at 48 h post-treatment (Figure 4). The polymer fragments of the auxin-treated samples at 48 h post-treatment were less depolymerized or more stable than those of the control and TIBA treatments and were similar to those of the control treatment at 0 h post-treatment (Figure 4). Thus, auxin could produce a contrasting effect on cell wall polymer stability with respect to ABA. In this line, we previously showed that strawberry fruits treated with ABA presented greater polymer fragmentation than the control sample [16].

### 2.3. Enzymatic Activity

Different authors have shown that auxin influences the activity of cell wall remodeling enzymes [34,60]. Auxin treatment induces expansin, pectin methylesterase (PME), xyloglucan endotransglycosylase/hydrolases (XTH) activity [60], and other enzymes.

**Figure 5:**
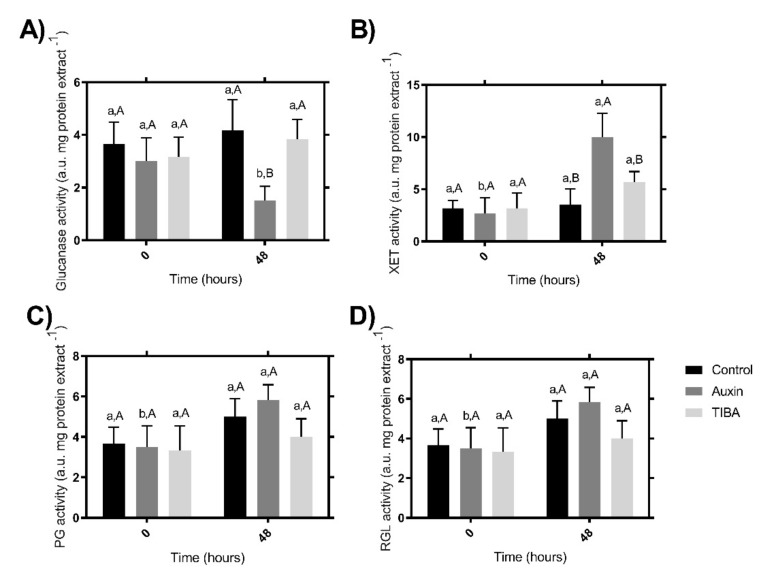
Patterns of total enzyme activities. Total glucanase/cellulase activity (**A**); total xyloglucan endotransglucosidase (XET) activity (**B**); total polygalacturonase (PG) activity (**C**); and total rhamnogalacturonan endolyase (RGL) activity (**D**). The enzymatic activities assayed during fruit treatments at 0 or 48 h post-treatment. Different letters indicate significant differences between cultivars and stages (*p < 0.05*).

As we have shown using TG analyses, there are differences in the stability and composition of the cell walls in fruits treated with auxin and TIBA after 48 h compared to the 0 h control. Various families of enzymes have been described as cell wall remodelers [18,27,28,29,30,31], and for this reason, we wanted to evaluate some of the most important families (Figure 5). Thus, to evaluate the putative enzymatic effect that explains the cell wall polymer degradation observed in the TGA curve (Figure 2B), we evaluated the enzymatic activity of the four principal groups of cell wall-related enzymes. To examine the relationships in the activities of the glucanase enzymes, the total glucanase activity was determined after auxin and TIBA treatments and contrasting with the control samples (Figure 5A). The lowest activity was detected after auxin treatment at 48 h post-treatment, while no significant differences were observed between the control- and TIBA-treated samples at the two times evaluated (Figure 5A). Additionally, as expected at time 0 h, no differences were observed between the three samples evaluated (Figure 5A).

The total XET activity showed an increase at 48 h post-auxin treatment, while no differences were detected between the other two treatments at the same time (Figure 5B). With respect to the PG and RGL total activity, no differences were found at 48 h post-treatment in the three sample groups (Figure 5C,D). Verma et al. (1965) [60] showed that auxin treatment in pea epicotyls induces cellulase activity in vivo and in vitro, leading to the cleavage of load-bearing hemicellulose chains. We believe that something similar could occur in the treated strawberry fruits, and for this reason, the total XET activity increases after 48 h post-treatment (Figure 5B), since auxin would release the hemicelluloses, making them available for the action of the enzymes with XET activity.

### 2.4. Evaluation of Changes in the Transcriptional Accumulation Level of Cell Wall-Related Genes

**Figure 6:**
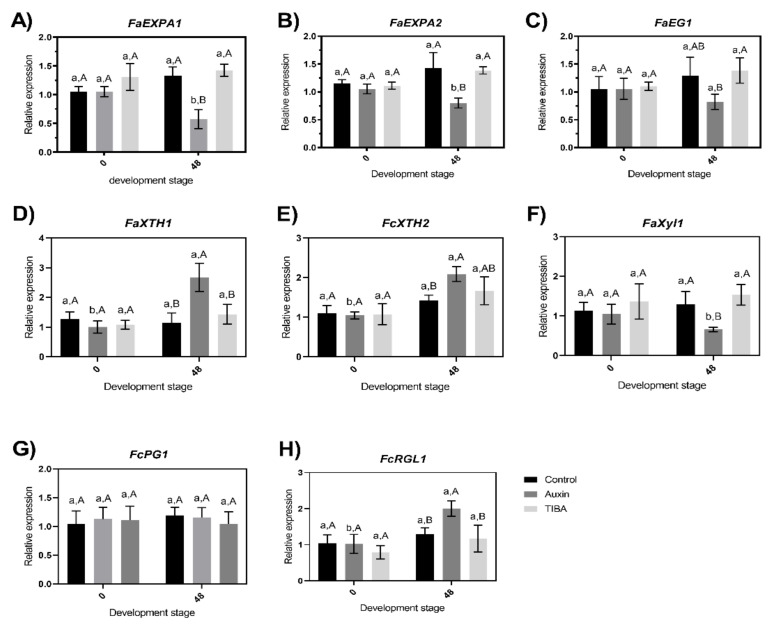
mRNA levels measured by RT-qPCR after auxin, TIBA, and control treatment of eight genes encoded by enzymes and proteins related with the cell wall remodeling. The expression data correspond to the mean of the three replicates normalized against *FaGADPH1* abundance. Different letters indicate significant differences between cultivars and stages (*p* < 0.05). The *F. x ananassa* genes correspond to: two *expansins A, FaEXPA1* (**A**) and *FaEXPA2* (**B**), one *endoglucanase* (*FaEG1*) (**C**), two *xyloglucan endotransglycosylase/hydrolases*: *FaXTH1* (**D**) and *FaXTH2* (**E**), one *β-xylosidase* (*FaXyl1*) (**F**), *polygalacturonase* (*FaPG1*) (**G**) and *rhamnogalacturonate lyase* (*FaRGL1*) (**H**).

The total enzymatic activity in the four cases represents the combined activities of several enzyme isoforms because cell wall-related enzymes are composed of a multigene family [16,49,61,62]. However, studying the complete family of each one of these genes seems not to be necessary when a small group of genes related to this multigene family has been described as molecular markers of fruit ripening, and more importantly, they are related to firmness decreases in strawberries [16,18,63]. Thus, the changes in the transcription level of eight genes that encode proteins or enzymes associated with the disassembly of the structural components of the cell walls during fruit ripening of strawberry fruits were evaluated (Figure 6). The selected genes have been previously studied and were found to respond to auxin treatment [2,11,33,34,63,64,65], and they showed different effects at the transcriptional level.

The transcriptional level of the eight genes analyzed here did not show significant differences at 0 h (Figure 6), indicating that the samples were selected under similar conditions at the cell metabolism level. Thus, the real effect of hormone treatment can be determined. The relative expression of the two alpha expansin genes (*FaEXPA1* and *FaEXPA2*) showed a decrease at 48 h after auxin treatment, and significant differences were observed between auxin-treated samples and TIBA- or control-treated samples at 48 h post-treatment or between the transcription levels of the 48 h auxin-treated samples and the auxin-treated samples at 0 h (Figure 6A,B). The activity proposed for the expansin proteins has the capacity to disrupt hydrogen bonds between hemicellulose and cellulose microfibrils in the cell walls [66,67]. These proteins are part of the first group of genes that increase their expression when fruit ripening advances [45]. For this reason, the decrease in the transcriptional level of these two expansin genes (Figure 6A,B) can explain the lower depolymerization of the auxin-treated samples observed in the TGA curve shown in Figure 2B. Furthermore, Valenzuela-Riffo et al. (2020) [15] recently showed that *FaEXPA5* did not show significant differences at 48 h post-treatment. Another important gene family that encodes enzymes that catalyze the initial attack to initial degradation on the cellulose polymer is the endo-1,4-β-glucanases; in particular, we evaluated *FaEG1* (an endo-1,4-β-glucanase), and significant differences were observed between auxin-treated samples and TIBA-treated samples at 48 h post-treatment (Figure 6C). This result was similar to that described by Chen et al. (2016) [32], who showed four different endoglucanases with negative log2 (fold change), indicating a decrease in the transcriptional level from strawberries treated with auxin.

A fruit-specific β-xylosidase enzyme is encoded by *FaXy1*, and this gene showed significant differences between auxin-treated samples with TIBA and control-treated samples at 48 h post-treatment (Figure 6F); the changes in the expression levels of *FaXyl1* are not new, and they are similar to those described by Bustamante et al. (2009) [47], who showed that the gene expression decreased after 72 h post-treatment with NAA (naphthalene acetic acid, a synthetic plant hormone in the auxin family), GA3 (gibberellic acid), and ethephon (an ethylene-generating compound). By contrast, *FaXTH1*, *FaXTH2*, and *FaRGL1* increased their transcriptional levels 48 h after auxin treatment (Figure 6D,E,H). Lastly, the effect at the *FaPG1* transcriptional level after TIBA or auxin treatment was not significantly different between the two treatments with respect to the control treatment samples (Figure 6G). *FaPG1* showed a contrasting gene expression level with respect to that described by Villareal et al. (2008) [65] using Northern blot analysis, showing that the *FaPG1* expression level was reduced in ‘Selva’ strawberries treated with auxin, and Villareal et al. (2009) [52] showed a similar reduced transcriptional level using Northern blot analyses after 72 h post-treatment in the same cultivar used in the present study (‘Camarosa’ strawberry). However, the technique used to evaluate the transcriptional level was less sensitive.

To understand why the auxin treatment produces differences in the expression levels of seven of the eight genes (Figure 6), we reviewed the literature for information on the regulatory regions of these genes and searched for putative *cis*-regulatory elements related to the primary hormones. First, for *FaXTH1* and *FaXTH2,* the promoter regions were described by Nardi et al. (2014) [68], and the authors identified at least one auxin response element in each promoter region. The promoter region of *FaXyl1* was described by Bustamante et al. (2009) [47] and showed the AuxRR-core motif that responds to auxin. *FaEG1*, the promoter region, was described by Spolaore et al. (2003) [69], and the auxin downregulating effect on GUS expression with the promoter of *FaEG1* was used, indicating that it responds to auxin [69]. The promoter regions of *FaEXPA2* and *FaPG1* were previously described by Castro et al. (2021) [16], and only *FaPG1* showed a cis-regulatory element related to auxin.

## 3. Materials and Methods

### 3.1. Plant Material

*F.× ananassa* ‘Camarosa’ fruits at the small white (SW) (19–20 days post-anthesis) stage were harvested from plants grown in a commercial orchard in Pelluhue, Maule Region, Chile (latitude 35°50′00″ S; longitude 72°38′00″ W). The harvest occurred during the 2019–2020 season (specifically in January 2020), and a total of 150–160 fruits were collected. The harvested fruits were immediately transported to the laboratory (Multidisciplinary Agroindustry Research Laboratory, Universidad Autónoma de Chile) under cold conditions.

### 3.2. Auxin and TIBA Treatments

Auxin (Aux) and 2,3,5-triiodobenzoic acid (TIBA) treatments were evaluated according to the methodology presented by Villarreal et al. (2009) [32]. In short, fruits in the small white (SW) stage (19–20 days post-anthesis) were randomly divided into three groups: (1) without Aux or TIBA treatment (as a control), (2) with Aux treatment, and (3) with TIBA. Each group contained thirty fruits. For hormone treatments, the fruits were dipped in hormone solution or water (as a control group 1) for 10 min in the appropriate solution. Group 2 was dipped in 100 μL of 1 mM IAA (3-indoleacetic acid, a synthetic auxin) (Sigma-Aldrich, St. Louis, MO, USA). Similarly, Group 3 was treated with 100 μL of 1 mM TIBA (Sigma-Aldrich, St. Louis, MO, USA). To prevent dehydration, the peduncle of each fruit was immersed in distilled water (see Figure S1). The fruits were maintained at 20 °C according to Villarreal et al. (2009) [52]. The samples were collected after 0 h and 48 h of treatment. Immediately after this collection, the calyx and peduncle were removed, and the treated fruits were dissected, frozen in liquid nitrogen, and stored at −80 °C until use. Complete random designs were used. The standard errors (SEs) and LSDs were calculated using the SPSS v.15 package at a significance level of 0.05.

### 3.3. Determination of Fruit Quality Parameters

The firmness was measured using a texture analyzer (model CT3, Brookfield Engineering Labs., Middleborough, MA, USA), weight was measured using semi-analytical balance (BEL model LW203I, Monza, MB, Italy), and the soluble solid contents (SSCs) were measured using a hand-held temperature compensated refractometer (Atago, Tokyo, Japan) and expressed as °Brix. The parameters were measured over fifteen treated and untreated harvest fruits according to the methodology implemented previously in the laboratory and described in Ramos et al. (2018) [18].

### 3.4. Evaluation of the Thermal Stability Using Thermogravimetric Analysis (TGA)

Thermogravimetric analysis (TGA) was realized using a Discovery SDT-650 thermogravimetric analyzer (TA instrument) according to the methodology previously standardized in the laboratory and employed in [16,33,54,70]. With respect to the samples, the thalamus from the five fruit treatments were homogenized with a mortar and pestle, and then, the macerated thalamus samples were dried at 80 °C for 48 h, and 10 mg of the dry sample groups were employed two times to determine the chemical characteristics of the degradation process. The dry samples were heated at a constant rate of 5 °C min^−1^ to temperatures between 50 and 500 °C in nitrogen.

### 3.5. RNA Isolation, Reverse Transcription, and RT-qPCR Analysis

RNA was isolated from the fruit samples two times post-treatment (0 h and 48 h) using the CTAB method [71] with modifications [18]. Three independent RNA extractions performed on each frozen pool of treated sample fruits. High-quality RNA without genomic DNA contamination was used for cDNA synthesis using a ‘First Strand cDNA Synthesis Kit’ (Fermentas Life Science, Glen Burnie, MD, USA) as previously reported by Ramos et al. (2018) [22]. The mRNA abundance of eight genes from *F. x ananassa*, namely two *expansins A* (*FaEXPA1* and *FaEXPA2*), two *xyloglucan endotransglycosylase/hydrolases* (*FaXTH1* and *FaXTH2*), one *polygalacturonase* (*FaPG1*), one *β-xylosidase* (*FaXyl1*), one *rhamnogalacturonate lyase* (*FaRGL1*), and *endoglucanase* (*FaEG1*), was measured by RT-qPCR analysis. The primers used for the RT-qPCR analysis are recorded in Table 1. Amplification reactions were performed according to Ramos et al. (2018) [18]. Each reaction was performed in triplicate (technical replicate) and normalized against the expression level of the *glyceraldehyde-3-phosphate-dehydrogenase* 1 (*FaGAPDH1*) gene, and for data analysis, we used the algorithm reported by Vandesompele et al. (2002) [72].

### 3.6. Total Enzymatic Activity Assays

The total rhamnogalacturonan endolyase (RGL), total xyloglucan endotransglycosylase (XET), total polygalacturonase (PG), and total glucanase (GLU) activities were measured according to Castro et al. (2021) [54]. The activity of the different enzymes was measured in triplicate using a pool of five fruits per sample, with two technical replicates.

## 4. Conclusions

To the best of our knowledge, this study is the first to investigate cell wall changes using TGA and DTG in strawberries or other fruits after auxin treatment. Thus, we showed that TGA and DTG analyses have potential as important tools for furthering our understanding of cell wall remodeling. TGA showed the differences in the percentage of degradation between the treated and untreated fruits with auxin, with the biggest differences shown in region II (Figure 2B), indicating that the treated fruit with auxin has the greatest thermal stability compared with the untreated and treated with TIBA fruits. Then, we showed that the cell wall disassembly process was retarded by auxin treatment. Therefore, our results support the hypothesis previously described by Chen et al. (2016) [32] that auxin delayed the ripening process of strawberries after harvest, and we present evidence that auxin is specifically related to the delay in the softening process of strawberry fruits.

## Figures and Tables

**Table 1:** Primers sequences (5′→3′) used in this study to real-time PCR (RT-qPCR).

Target Gene	Accession Number	Primer Forward/Reverse
*FaEXPA1*	AF163812	5′-AACTTCTGCCCTCCCAACTT-3′
		5′-TGAACCTGATCCCACCCTTC-3′
*FaEXPA2* ^a^	AF159563	5′-CCGAGTTACTATTTGCGGTGA-3′
		5′-CACGTTGCCTCTCCCTAATC-3′
*FaXTH1* ^b^	GQ367550	5′-ACTCTGCTCTTGAGCATAGTGC-3′
		5′-GAGCTGAATCTCATTGCCACC3-3′
*FaXTH2* ^b^	GQ367551	5′-AGCTTTCTTTTGGGTTCTCTCTC-3′
		5′-CCTTAACAACCAAAGCAGATGGT-3′
*FaPG1* ^a^	AY282613	5′-CGCCTCTTGCTTGTGCTAC-3′
		5′-TCACACTGCATTGATCTCACC-3′
*FaEG1* ^c^	AJ414709	5′-CCACGGGCTCTATCAAAATC-3
		5′-TGGCCTTCGAAGAAGAGG-3′
*FaRGL1* ^d^	CO381780.1	5′-TCCCTGATCGCTCAGCTGCCGA-3′
		5′-TCGTGAGAGTTGGATCCTCGTGCCG-3′
*FaXyl1*	AY486104	5′-ATGTACAATGGAGGCCAAGC-3′
		5′-GCCATTCCAATTGTCGAGAT-3′
*FaGAPDH1* ^b^	AB363963	5′-TCCATCACTGCCACCCAGAAGACTG-3′
		5′-AGCAGGCAGAACCTTTCCGACAG-3′

^a^ The specific primers to FaEXPA2 and FaPG1 were obtained from Ramos et al. (2018) [18]. ^b^ The specific primers to FaXTH1, FaXTH2, and FaGAPDH1 were obtained from Nardi et al. (2014) [68]. ^c^ The specific primers to FaEG1 were obtained from Jara et al. (2019) [46]. ^d^ The specific primers to FaRGL1 were obtained from Molina-Hidalgo et al. (2013) [63].
